# As Others See Us

**Published:** 1899-10

**Authors:** 


### As Others See Us.

Professor Mosso, of Turin, the eminent Italian physiologist, has been visiting this country, and, according to the Rome correspondent of the *Lancet*, has sent home some of his impressions. Speaking of the physical development of Americans he says, in the letter quoted: "It is enough to look at the passers-by in the American streets to be convinced how much more developed and strong they are than our compatriots. The boys and girls in point of physique are far superior to ours. . . . America teaches us by the plainest and the most impressive of examples that physical education may be carried to perfection without any military object." It has been so much the rule with us to consider the average American physique as not quite what it should be, that it is refreshing to have such testimony from a competent foreign observer. It is probable that we have been given too much to self-depreciation; it has been as much too common to underestimate ourselves in some respects as to glorify ourselves in others. The average American may be less plump than his British fellow Anglo-Saxon—if it is correct to call him so—but in bone and muscle and nerve he is in all probability at least his equal, if not, taking all classes together, his superior. This is, of course, mentioned only as an anthropologic fact.—*The Journal.*

By the will of the late Dr. Chas. J. Stille, of the University of Pennsylvania, the department of political economy of Yale is to receive $75,000.

